# Peptide enzyme‐linked immunosorbent assay (pELISA) as a possible alternative to the neutralization test for evaluating the immune response to IBV vaccine

**DOI:** 10.1186/s12917-021-02757-5

**Published:** 2021-01-25

**Authors:** Qi Wu, Zhixian Lin, Jinsen Wu, Kun Qian, Hongxia Shao, Jianqiang Ye, Aijian Qin

**Affiliations:** 1 Key Laboratory of Avian Preventive Medicine, Ministry of Education, Yangzhou University, 12 East Wenhui Road, 225009 Yangzhou, Jiangsu P.R. China; 2 Jiangsu Key Lab of Zoonosis, 12 East Wenhui Road, 225009 Yangzhou, Jiangsu P.R. China; 3 The International Joint Laboratory for Cooperation in Agriculture and Agricultural Product Safety, Ministry of Education, Yangzhou University, 12 East Wenhui Road, 225009 Yangzhou, Jiangsu P.R. China; 4 Jiangsu Co-innovation Center for Prevention and Control of Important Animal Infectious Diseases and Zoonoses, 12 East Wenhui Road, 225009 Yangzhou, Jiangsu P.R. China; 5 Ministry of Education Key Lab for Avian Preventive Medicine, Yangzhou University, No. 12 East Wenhui Road, 225009 Yangzhou, Jiangsu P.R. China

**Keywords:** Infectious bronchitis virus, Peptide ELISA, Neutralizing antibody, Evaluation

## Abstract

**Background:**

Infectious bronchitis virus (IBV), a coronavirus, is one of the most important poultry pathogens worldwide due to its multiple serotypes and poor cross-protection. Vaccination plays a vital role in controlling the disease. The efficacy of vaccination in chicken flocks can be evaluated by detecting neutralizing antibodies with the neutralization test. However there are no simple and rapid methods for detecting the neutralizing antibodies.

**Results:**

In this study, a peptide enzyme-linked immunosorbent assay (pELISA) as a possible alternative to the neutralization test for evaluating the immune response to IBV vaccine was developed. The pELISA could indirect evaluate neutralizing antibody titers against different types of IBV in all tested sera. The titers measured with the pELISA had a coefficient of 0.83 for neutralizing antibody titers.

**Conclusions:**

The pELISA could detect antibodies against different types of IBV in all tested sera. The pELISA has the potential to evaluate samples for IBV-specific neutralizing antibodies and surveillance the infection of IBV.

## Background

Infectious bronchitis (IB) is a highly contagious disease caused by infectious bronchitis virus (IBV). IBV belongs to the *Coronaviridae* family, which includes severe acute respiratory syndrome coronavirus (SARS-CoV), Middle East respiratory syndrome coronavirus (MERS-CoV) and the recently emerged novel human coronavirus SARS-CoV-2 [1, 2]. IBV infection causes serious respiratory and renal diseases in meat chickens, egg-laying drops and false layer syndrome in laying hens, increasing secondary infections / processing plant condemnations and resulting in substantial economic losses in the poultry industry [3–5]. Although vaccines are now being used widely and extensively, the epidemic of IB in chicken flocks can still be observed [6]. How can the efficiency of a vaccine in immunized chicken flocks be evaluated? Generally, the detection of neutralizing antibody titers by cell culture is the best method. However, this process is time consuming and laborious and requires limiting the number of samples rather than performing large-scale sample detection [7, 8]. Are there faster and easier ways to determine the titers of serum neutralizing antibodies (Abs)? Several well-known serological techniques, including immunofluorescence [9] and enzyme-linked immunosorbent assay (ELISA) [10], have been tested to potentially replace the neutralization test for other viruses. Recent studies have shown that rabies virus and bovine viral diarrhea virus glycoprotein serology ELISAs can measure the titers of neutralizing antibodies in sera from vaccinated humans and cattle, respectively [11, 12]. The correlation of antibody titers between indirect ELISA and neutralization tests has also been studied in Zika virus and human papillomavirus [13, 14].

ELISA for detecting antibodies against IBV have been developed with the whole virion or recombinant S1 proteins, N proteins and nonstructural proteins [15–17]. These ELISA methods have achieved good results in detecting IBV antibody. However these methods could not evaluate the neutrolization antibody level in immunized chickens. To date, serological alternatives to neutralization tests for IBV have not been studied.

The IBV genome encodes four major structural proteins, spike (S), small envelope (E), membrane (M) and nucleocapsid (N); fifteen nonstructural proteins; and some accessory proteins [18]. Among these proteins, the S glycoprotein is thought to be the major protective antigen carrying neutralizing epitopes that can induce efficient immune responses against IBV [19–21]. S glycoprotein is cleaved by a furin-like host cell protease, generating the subunits of S1 and S2, respectively. Both S1 and S2 play a key role in vaccine development and serological methods. However, S1 is highly variable among different IBV stains, even though it is a major protein that induces protective antibodies against IBV [22, 23]. In contrast to S1, S2 is a highly conserved protein and carries broad antigenic epitopes [24]. Some neutralizing epitopes have been identified in the S2 protein [25, 26]. Our previous studies also demonstrated that an epitope in S2 was a broad-spectrum neutralizing epitope and showed that a key amino acid determined the broad spectrum of this epitope [27]. In this study, the pELISA with the peptide (SCPYVSYGRFCIQPDGSIKQ) in the S2 protein of CK/CH/2010/JT1 was compared with the neutralization test specific for IBV in serum samples. The possible alternative of the pELISA to the neutralization test for evaluating the immune response to IBV vaccine was discussed.

## Results

### Effect of the pELISA

**Fig. 1:**
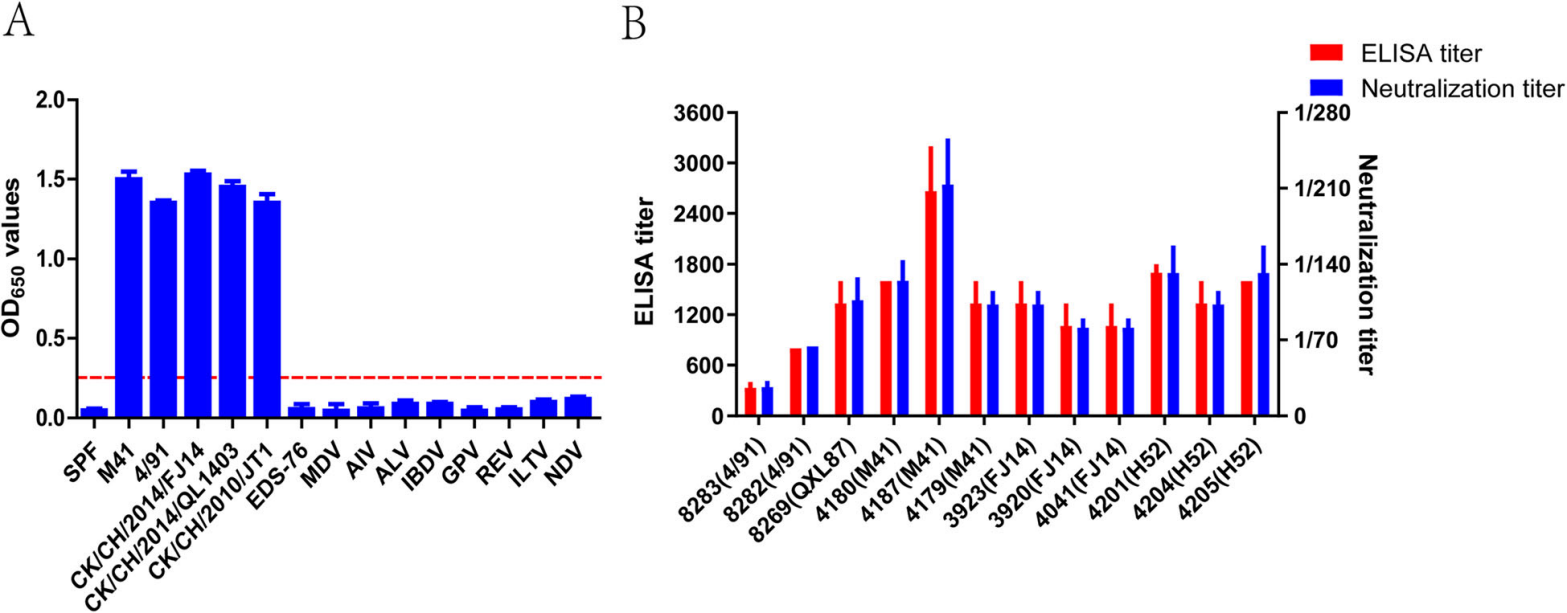
Evaluation of the pELISA. **a** Specificity of the pELISA. The red horizontal dotted line indicates the cut-off value. EDS-76, AIV, NDV, ALV, IBDV, GPV, REV, and ILTV. **b** Correlation between the pELISA titer and neutralization titer. Notes: The ELISA titer means the maximum dilution of the serum sample with OD value greater than the pELISA’s cut-off. Serum samples 8282 and 8283 were the immune sera of the 4/91 vaccine strain; serum sample 8269 was the immune serum of the QXL87 vaccine strain; serum samples 4179, 4180 and 4187 were the immune sera of the M41 virulent strain; serum samples 3920, 3923 and 4041 were immune sera of the CK/CH/2014/FJ14 virulent strain; and serum samples 4201, 4204 and 4205 were immune sera of the H52 vaccine strain

To evaluate the pELISA, the specificity and reproducibility of pELISA was detected. The results showed that immune sera against other viruses, such as NDV, ALV, MDV, AIV, IBDV, GPV, REV, ILTV and EDS-76V, were negative in the pELISA (Fig. 1). And the inter-assay and the intra-assay coefficient of variation of pELISA were less than 10 %.

To further evaluate the pELISA, 300 field serum samples were tested and analyzed with the pELISA and IFA. The results showed that 268 and 32 serum samples were classified as positive and negative, respectively, by the pELISA, while 271 and 29 serum samples were classified as positive and negative, respectively, when the same samples were analyzed by the IFA (Table 1). Compared to the IFA, the pELISA showed 98.15 % sensitivity, 93.1 % specificity and 97.76 % accuracy for the 300 serum samples, respectively.

**Table 1:** Comparison of the peptide ELISA with an IFA

IFA	pELISA		
	Positive	Negative	Total
Positive	266	5	271
Negative	2	27	29
Total	268	32	300

### Comparing the ELISA titers and neutralization titers of immune sera against different types of IBV

To evaluate the correlation between the pELISA and neutralization assays, twelve immune serum samples against 4/91, M41, H52, the QXL87 vaccine strain, and the CK/CH/2014/FJ14 virulent strain were evaluated. As shown in Fig. 1b, the pELISA titers of the serum samples 8269 (QXL87), 4179 (M41), 3923 (CK/CH/2014/FJ14) and 4204 (H52) were 1333, while the neutralization titers of these samples were 1:102. The ELISA titers of the M41 serum samples 4179, 4180 and 4187 were 1333, 1600 and 2666, respectively, while the neutralization titers were 1:102, 1:124 and 1:213, respectively. The results indicated that the ELISA titers of the tested serum samples had a positive correlation with the neutralization titers.

### Time course of serum ELISA and neutralizing antibody levels following IBV infection and vaccination

**Fig. 2:**
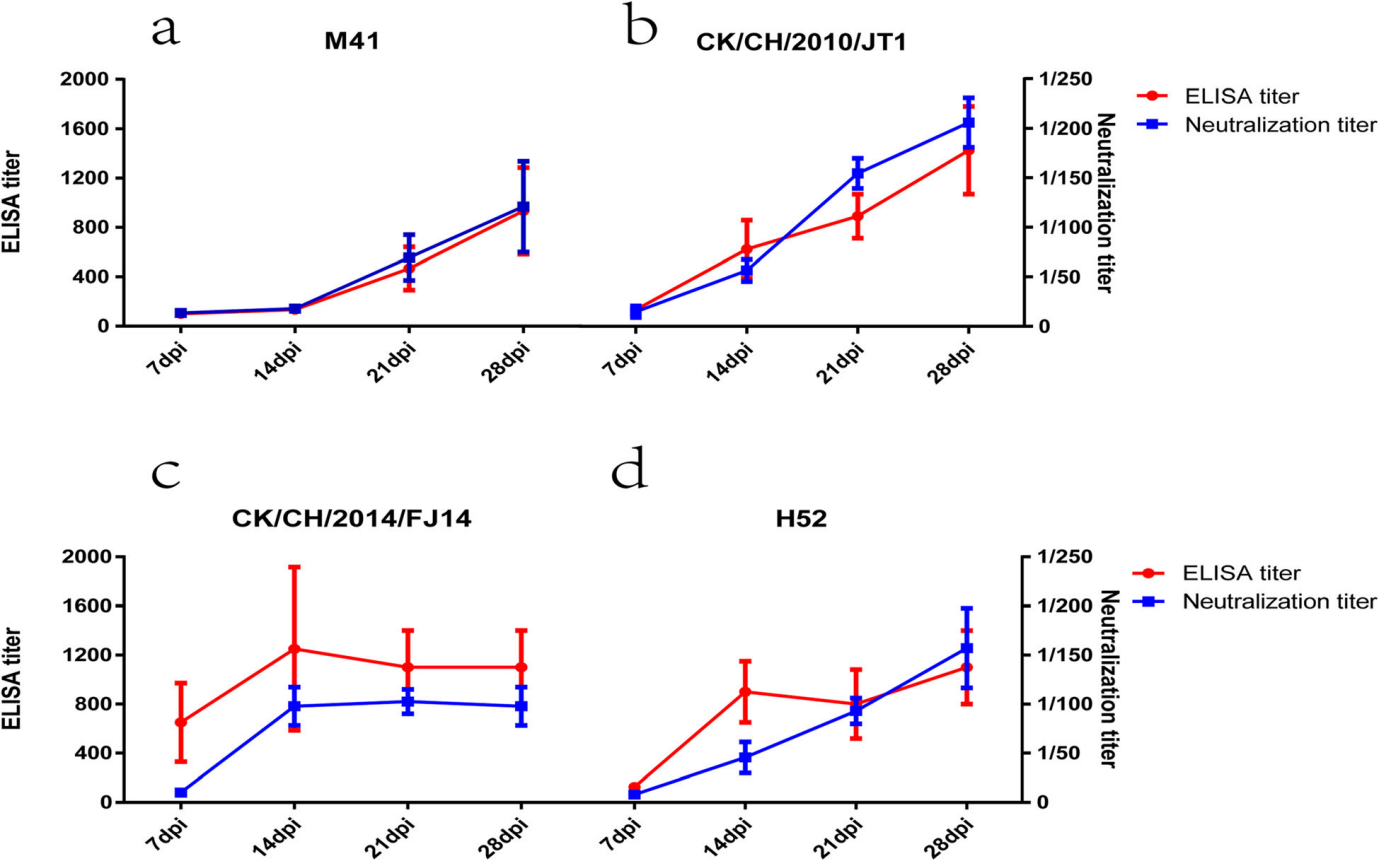
The correlation between the ELISA titer and neutralization titer for serum antibodies against IBV. Comparison of the titers measured by pELISA and a neutralization assay for chicken sera against CK/CH/2010/JT1 (**a**), CK/CH/2014/FJ14 (**b**). M41 (**c**), and the H52 attenuated vaccine (**d**). The ELISA titer means the maximum dilution of the serum sample with OD value greater than the pELISA’s cut-off. There were five serum samples evaluated at each time point, and each serum sample was independently tested three times

To further evaluate the pELISA, 14-day-old SPF chickens were infected with the IBV CK/CH/2014/FJ14 strain, CK/CH/2010/JT1 strain or M41 strain or vaccinated with the H52 strain. Serum samples were collected from the infected or vaccinated chickens on different days post infection, and serum ELISA titers were measured by pELISA. Then, the results were compared with the neutralization titers measured in the neutralization assay. As shown in Fig. 2, the ELISA titers of the serum samples collected from the chicken post inoculation with CK/CH/2010/JT1 on days 7, 14, 21, and 28 were 1:100, 1:466.6, 1:666.6, and 1:1066, while the neutralization titers of these serum samples were 1:14.8, 1:56.25, 1:154.5, and 1:206 (Fig. 2b). The ELISA titers and neutralization titers of sera increased as the time after infection increased, and good concordance between the ELISA titers and neutralization titers was observed in the tested sera collected at the different points post inoculation with M41 or CK/CH/2010/JT1 (Fig. 2 a and b). Similarly, there was also a very distinguished positive correlation between the ELISA titers and neutralization titers in chicken sera collected post infection with the CK/CH/2014/FJ14 virulent strain, even the serum samples at 7 days after infection had some differences between ELISA titers and neutralization titers (Fig. 2c). In the serum samples from chickens inoculated with the H52 vaccine, the overall positive correlation was excellent, although there was some difference between the ELISA titer and neutralization titer at time point 14 days after inoculation (Fig. 2d).

## Discussion

**Fig. 3:**
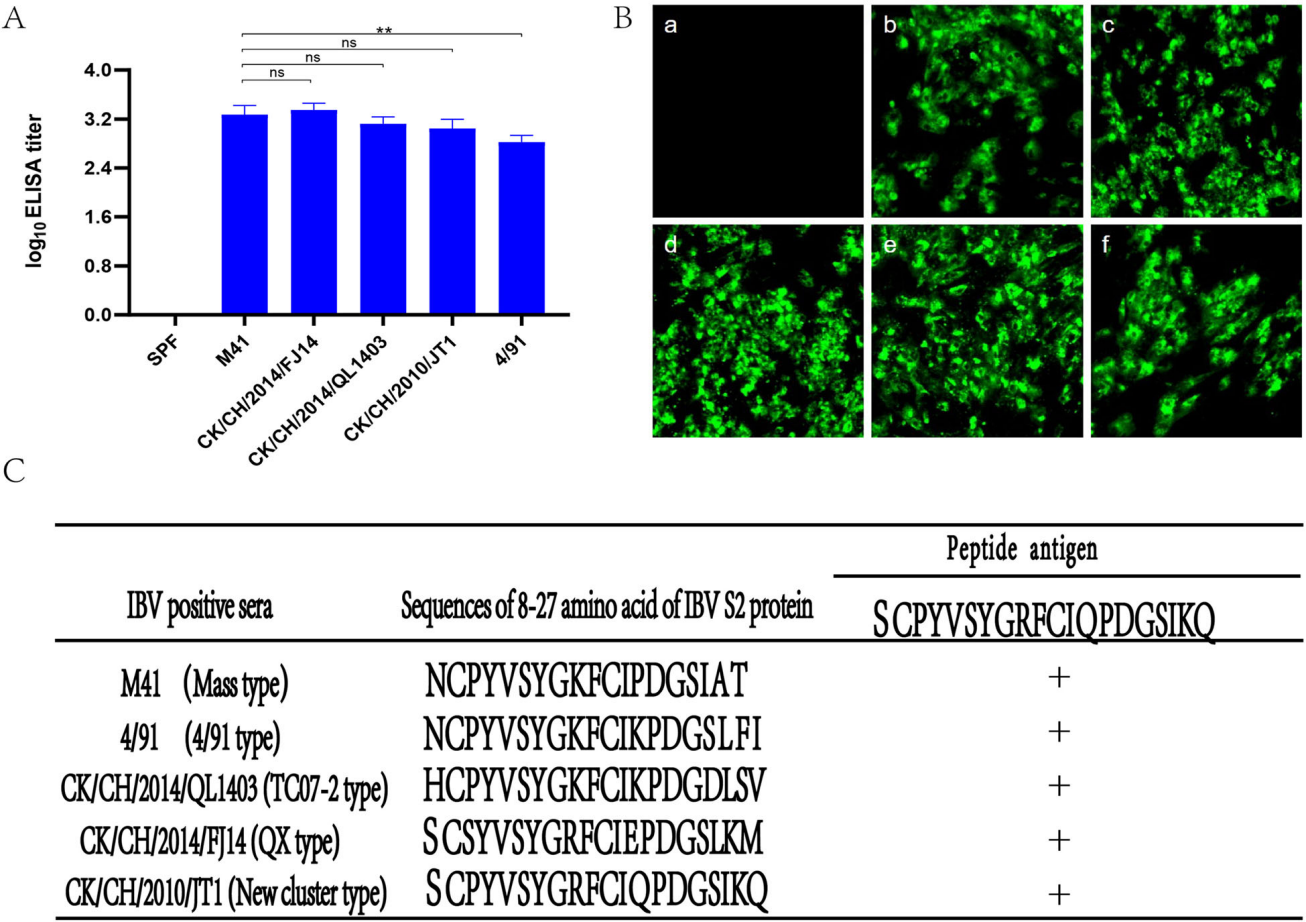
Reactivity of the peptide with immune sera against different genotypes of IBV strains. (A) Reaction with immune sera against different IBV strains. The ELISA titer means the maximum dilution of the serum sample with OD value greater than the pELISA’s cut-off. ** *p*<0.01; ns (nonsignificant); *p *>0.05. (B) These sera were proven to be positive against IBV by an IFA. a, represents SPF chicken serum; b, c, d, e, and f represent M41 immune serum (Mass-type), 4/91 serum (4/91-type), CK/CH/2014/QL1403 serum (TC07-2-type), CK/CH/2014/FJ14 serum (QX-type), and CK/CH/2010/JT1 serum (New cluster-type), respectively. (C) Comparative amino acid sequence analysis of the peptides used for pELISA and the vaccine and reference strains used for immune serum production. “+” represented the positive serum against corresponding to strain could react with the peptide

IBV has been one of the most important pathogens threatening chicken flocks and causes substantial economic losses in the poultry industry worldwide [28]. Vaccination and monitoring of antibody levels in immunized chicken flocks are important for the prevention and control of IBV infection [29–32]. To detect IBV-specific antibodies as accurately and comprehensively as possible, many ELISA-based antibody detection methods have been established [33–35]. Such as, Ding et al. developed a multi-fragment antigen ELISA showed good coincidence ratio with the commercial ELISA (IDEXX) [36]. Lei et al. established nsp5-based ELISA revealed consistent with the commercial ELISA in detecting IBV specific antibody levels following IBV infection and vaccination [16]. However, these methods and commercial ELISAs could only monitor the antibody levels of immunized chicken flocks. Most of these methods cannot evaluate the protective effect or neutralizing antibody level. Hemagglutination inhibition (HI) with an IBV antigen showed some relation to the neutralizing antibody titer, but it was sometimes not very stable. In the present study, a pELISA method was established by using a synthetic peptide derived from the identified broad-spectrum epitope. There are two cysteines in the peptide. It could increase the possibility of peptide cyclization by disulfide bonding between two Cys residue, which may make the peptide more stable. The pELISA is a simple, rapid, sensitive and broad-spectrum method. Our results demonstrated that the pELISA had a good reaction with immune sera against different types of IBV (Fig. 3).

Vaccination is an effective means to control and prevent IBV infection [29]. However, the continued mutation and recombination of the IBV genome and poor cross-protection between different types of IBV have put great pressure on the development of IBV vaccines [28, 29]. Evaluation of vaccine efficacy includes measuring the neutralizing antibody titer, survival rate, morbidity, tissue lesion, and viral load. The neutralizing antibody titer is the most critical parameter [37, 38]. The neutralizing antibody titer is primarily determined by neutralizing assays [2, 39]. Although neutralization tests accurately reflect the neutralizing antibody titers in the serum, the technique is time consuming and laborious, which are inevitable weaknesses [40, 41]. In the present study, pELISA with a broad-spectrum epitope not only react with different genotype IBVs sera but also indirectly reflect the neutralizing antibody levels of immune sera against IBV. The correlation coefficient between ELISA titers and neutralization titers was 0.90, 0.88, 0.81 and 0.64 for the sera against Mass-type virulent strain (M41), the new cluster genotype strain (CK/CH/2010/JT1), and QX vaccine and virulent strains (QXL87 and CK/CH/2014/FJ14) and H52, respectively. The possible reasons for the correlation deviation/variations between pELISA titer and neutralization titer of different strains may be the amino acid variations on S2 protein of different IBV strains. In short, we found that the total correlation coefficient was 0.83. We think that the pELISA could potentially be used to evaluate neutralizing antibody level against IBV. It could be better used for tracking vaccination and exposure of IBV in the field even different strain shows different neutralization antibody level or protection against one particular type of virus (Serotype).

Currently, ELISA has been widely used in neutralizing antibody detection to replace neutralizing assays for many different viruses due to its simplicity, rapidness, sensitivity and suitability for large-scale use [12–14]. Zhao et al. demonstrated that a RABV GP protein-based ELISA could be a suitable method for measuring neutralizing antibody titers in human serum samples to assess the vaccination status[12]. Similarly, for the measurement of neutralizing antibodies against human papillomavirus, a VLP-based ELISA was an acceptable surrogate for a neutralizing antibody assay in measuring vaccine responses [14]. The serological outcomes of a Zika virus envelope protein-based ELISA also correlated with the ZIKV neutralization capacity measured in vitro [13]. Several ELISA techniques proposed to replace the neutralization test for detecting neutralization-relevant antibodies to polioviruses might offer an alternative to the neutralization test [8]. As for the coronavirus, S glycoprotein is surface exposed and mediates entry into host cells, it is the main target of neutralizing antibodies upon infection and the focus of therapeutic and vaccine designs [28, 42, 43]. Our results above show that pELISA can detect serum antibodies against different types of IBV strains and the the correlation coefficient between neutralization titers and ELISA titers is excellent. The time course of IBV antibody detection by pELISA indicates that pELISA has the potential to replace neutralization assays for evaluating the effects of vaccines.

## Conclusions

A pELISA as a possible alternative to the neutralization test for evaluating the immune response to IBV vaccine was developed. The pELISA could indirect evaluate neutralizing antibody level against different types of IBV in all tested sera. The titers measured with the pELISA had a coefficient of 0.83 for neutralizing antibody titers.

## Methods

### Virus and serum samples

The M41 strain (GenBank accession number: DQ834384) and H52 strain (GenBank accession number: EU817497) of IBV were obtained from Sinopharm Yangzhou Vac Biological Engineering Co., Ltd. (Yangzhou, China). The CK/CH/2010/JT1 (GenBank accession number: KU361187), CK/CH/2014/FJ14 (GenBank accession number: MN262521) and CK/CH/2014/QL1403 (GenBank accession number: KU361198) strains of IBV were isolated and identified by our laboratory [27]. Immune serum against QXL87 (GenBank accession number: MH743141) vaccine strain (QX-type) was obtained from Zhongchong Sino Biological Technology Co., Ltd (Shanghai China). Sera against the M41, H52, CK/CH/2010/JT1, CK/CH/2014/QL1403, and CK/CH/2014/FJ14 strains were obtained according to our previous preparation methods [27]. Reference negative sera were collected from SPF chickens, which were confirmed to be free of IBV antibodies by an immunofluorescence assay (IFA). Three hundred field serum samples vaccinated with vaccine H120 strain in 14 days of age were randomly obtained from chicken flocks in different areas of Jiangsu Province, China during 2017–2018. All the experiments complied with institutional animal care guidelines were approved by the University of Yangzhou Animal Care Committee (Authorized XYSK (Su)2016-0020). We have acquired a permission from Yangzhou University to collect animal samples.

### pELISA procedure

The epitope was synthesized by Synpeptide Co., Ltd. (Shanghai, China), and pELISA was preformed as our previous paper [27].

### pELISA specificity and reproducibility

The specificity of the pELISA was tested with serum samples positive against avian influenza virus (AIV), avian leukosis virus (ALV), reticuloendotheliosis virus (REV), gosling plague virus (GPV), Newcastle disease virus(NDV), infectious bursal disease virus(IBDV), infectious laryngotracheitis virus(ILTV), Marek’s disease virus(MDV), or egg drop syndrome-76 virus(EDS-76). The reproducibility within and between runs was assessed as described above with eight serum samples (4 positive samples and 4 negative samples evaluated by indirect immunofluorescence). The mean OD_650_ value, standard deviation (SD) and coefficient of variation (CV) were calculated. The calculation formula of Coefficient of variation (CV) is standard deviation (SD)/ mean×100 %.

### Determination of the serum neutralization titer

All sera were heat inactivated at 56 °C for 30 min, two-fold serially diluted and incubated with the same volume of 100 50 % tissue culture infectious dose (TCID_50_) of the IBV strains (M41 strain, 4/91 strain, CK/CH/2014/FJ14 strain and CK/CH/2010/JT1 strain) at 37 °C for 1 h. The mixtures were then added to 96-well plates containing 80 % confluent CEK cell monolayers and incubated for 2 h at 37 °C in 5 % CO_2_. The supernatant was removed, 200 µl of DMEM/F12 supplemented with 2 % FBS was added, and the plates were incubated for 48 h. The cells were fixed and examined by an indirect immunofluorescence assay. The neutralizing titer of each serum sample against IBV was determined and calculated by the Reed and Muench method.

### Indirect immunofluorescence assay (IFA)

The IFA was performed as previously study [44]. Briefly, Virus-infected and uninfected cells were fixed in a 3:2 v/v mixture of acetone and ethanol and washed once with PBS. The fixed cells were incubated with the sera against IBV. After three washes with PBS, the cells were incubated with the FITC-conjugated goat anti-chicken IgG antibody (Jackson ImmunoResearch Laboratories, Inc; Jackson, USA). After three washes with PBS, the cells were observed under a fluorescence microscope.

### Statistical analysis

All the data were statistically analyzed with Prism 5 software (GraphPad, La Jolla, CA). One-way ANOVA with repeated measures was used to evaluate the reactivity of peptide antigens with immune sera against IBV. Differences were considered statistically significant at *p* values < 0.01.

## Data Availability

The datasets generated and/or analyzed during the current study are available in the GenBank accession number: DQ834384, KU361187, MN262521, KU361198 and MH743141.

## Abbreviations

IBV: infectious bronchitis virus
pELISA: peptide enzyme-linked immunosorbent assay
IFA: Indirect immunofluorescence assay
DMEM/F12: Dulbecco’s modified Eagle’s medium nutrient mixture F-12
BSA: Bovine serum albumin
FBS: fetal bovine serum
FITC: Fluorescein isothiocyanate
CEK: Chicken embryonic kidney
